# The influence of grit on life satisfaction of Brazilian undergraduate students: academic adaptation as a mediator

**DOI:** 10.3389/fpsyg.2024.1331259

**Published:** 2024-04-02

**Authors:** Ana Paula Porto Noronha, João Lucas Dias-Viana, Ana Paula Ozório Cavallaro

**Affiliations:** Departament of Psychology, Postgraduate Program in Psychology, Universidade São Francisco, Campinas, São Paulo, Brazil

**Keywords:** college students, grit, higher education, soft skills, life satisfaction

## Abstract

**Introduction:**

In recent years, research in educational contexts has pointed to the role of grit as a variable related to various positive outcomes, including life satisfaction. Academic adaptation seems vital for the success of academic life in college students. Considering university as an important life context for people pursuing higher education, what is the mediating effect of academic adaptation? This study proposed a mediation model to examine the mediating effect of academic adaptation in the relationship grit and life satisfaction.

**Methods:**

A sample of 413 undergraduate Brazilian students, age ranging from 18 to 71 years (*M* = 27.30; SD = 10.20). A correlation analysis was performed between the variables and the following mediation model was tested: Grit (independent variable), academic adaptation (mediating variables) and life satisfaction (dependent variables).

**Results:**

Mediation analysis indicated that academic adaptation mediated around 46.80% for the factor of consistency of interests and 40.90% of the relationship with perseverance of effort. Thus, the greater the grit of the university student, the greater the grit, which leads to better satisfaction with life.

**Discussion:**

In recent years, research in educational contexts has pointed to the role of grit as a variable related to various positive outcomes. The findings elucidated that grit in academic context could benefit students’ adaptation process, and the university’s responsibility to improve its students’ softskills, not only for the future stage after university, but also during the student’s schooling.

## Introduction

The study of psychological resources for achieving good academic results has attracted the attention of researchers around the world, beyond the cognitive capacity of individuals (Duckworth et al., 2007; Credé et al., 2017). Grit is a personality trait (defined as consistency of interest and perseverance of effort) (Duckworth et al., 2007) that is necessary for achievement in various areas of life (Khan and Khan, 2017; Cormier et al., 2019; Mosewich et al., 2021), including academic (Casali et al., 2023). There is evidence that grit is positively associated with life satisfaction (Khan and Khan, 2017), that is the cognitive dimension of subjective well- being and refers to the people’s global evaluation of the quality of their life (Diener et al., 1997). This relationship can be direct (Singh and Jha, 2008), mediated, or moderated by other variables (Li et al., 2018). In this paper, in addition to grit, academic adaptation, which is the capacity of the college students to effectively adjust the changes imposed by new educational environment, is investigated, especially in relation to its mediating effect on the relationship between grit and life satisfaction (Collie et al., 2016; Mendes, 2021). Despite the growing interest in the relationship between grit and life satisfaction, few studies have investigated this dynamic in Brazilian students, especially when the academic adaptation variable is included. This research seeks to fill this gap, as positive constructs can be protective of challenging situations such as those found in higher education, such as greater demands, separation from family members, adaptation to different rules, and establishment of new interpersonal relationships.

Grit is a multifactorial construct that includes the dimensions of constant interest and perseverance to achieve a long-term goal. Perseverance of effort pertains to the degree to which individuals exert long-lasting determination in confronting obstacles. And consistency of interest relates to the propensity to embrace a consistent range of interests over a prolonged duration. Grit is one of the psychological variables that can help to answer the question: “why do some individuals accomplish more than others with equal intelligence?”. As grit is observed in behaviors manifested by the ability to sustain effort and interest in long-term goals, even in the face of adversity (Noronha and Almeida, 2022). Grittier people seem to face their fails as commas and not final points, meaning that they will continue to make an effort on that specific goal they have even if it is tough, they will persevere and be passionate about their long term goals (Duckworth et al., 2007; Duckworth and Quinn, 2009).

Duckworth et al. (2007) made the initial theoretical proposition of the construct, which subsidized the development of the Grit Scale (Duckworth et al., 2007; Duckworth and Quinn, 2009), and other similar scales to measure the construct in different populations and cultures (Disabato et al., 2018; Clark and Malecki, 2019; Datu and Zhang, 2020; Postigo et al., 2020; Singh and Chukkali, 2021). These instruments prompted researchers around the world to investigate the relationship that grit establishes with other variables (Li et al., 2018; Tang et al., 2019; Alhadabi and Karpinski, 2020; Ain et al., 2021; Casali et al., 2023).

Studies indicate that grit has a positive relationship with life satisfaction (Singh & Jha, 2008; Datu et al., 2016; Credé et al., 2017; Li et al., 2018; Ain et al., 2021). Life satisfaction refers to the cognitive component of subjective well-being, i.e., a person’s perception of how satisfied they are with their life. Subjective well- being is a tripartite construct, made up of cognitive and affective dimensions (positive and negative affections) (Diener et al., 1997). Life satisfaction appears to be more stable than the affective components, however, it can be affected by some factors, namely external events (Anusic and Schimmack, 2016), age (Fergusson et al., 2015), gender (Chen et al., 2019) and personality traits (Casali et al., 2023), such as grit. According to Khan and Khan (2017) individuals who are determined and committed to achieving ambitious and long-term goals, regardless of the difficulties encountered, are generally more satisfied with themselves and their lives. Moreover Casali et al. (2023) argues that grit functions as an internal resource enabling growth in the face of adversity which in turn can lead to greater satisfaction in the lives of individuals.

Results from a study of 776 North American adolescents found a correlation of r = 0.41 between the constructs (Clark and Malecki, 2019). The relationship between grit and life satisfaction is not always direct, and can be mediated by other variables (Li et al., 2018; Oriol et al., 2020; Casali et al., 2023). A study of 243 Chinese workers found correlations ranging from 0.28 to 0.47, magnitudes classified as low to high. In addition, the results indicated the direct effect of grit on life satisfaction, as well as optimism as a mediating variable between the constructs (Li et al., 2018). Thus, just as important as external variables, personality traits, which are more stable, are fundamental in influencing how people perceive and evaluate their lives. In a meta-analysis a significant and positive relationship was identified between grit and life satisfaction. Specifically, the perseverance of effort dimension yielded higher results (*p* = 0.54) compared to the consistency of interest dimension (*p* = 0.20).

In addition to its individual benefits, grit is considered an important non-cognitive variable in educational contexts due to its direct effects on academic outcomes, such as school performance (Duckworth et al., 2007; Credé et al., 2017; Clark and Malecki, 2019; Alhadabi and Karpinski, 2020), school permanence (Eskreis-Winkler et al., 2016), school engagement. It should be highlighted that the study of grit is relatively recent (Duckworth et al., 2007) and that other important variables for the field of school psychology should be investigated in order to better understand their relationship with grit. The relationship between these studies is justified because of the contribution of their findings so that effective interventions can be planned for the educational context (Vainio and Daukantaite, 2016).

In this sense, in the academic context, another variable to be considered and its relationship with grit studied is academic adaptation, which refers to the ability of students to adjust effectively to the demands of the educational environment. The construct encompasses the extent to which students are able to develop learning strategies, deal with stressful situations and respond to academic challenges in a constructive way, in addition to creating a network of relationships (Almeida, 2007; Nadelson et al., 2013). Academic adaptation is crucial for academic success, as students who are able to adapt to new learning environments and overcome obstacles are more likely to achieve their academic goals (Collie et al., 2016). Academic adaptation is the way students deal with adversity in the academic environment (Chen et al., 2023). It is a multidimensional process, encompassing various aspects to be considered (e.g., particularity of the course, culture and climate of the educational institution, learning process, and academic success), requiring the individual to achieve adaptive skills in the face of the demands of the new context (Mendes, 2021; Chen et al., 2023). In this way, grit seems to be an important characteristic for students in their training process, whether at school or university, since the construct refers to persistence in effort (even in the face of adversity, the individual will persist to achieve their goals) and consistency of interest (staying interested and focused) (Duckworth et al., 2007; Noronha and Almeida, 2022).

There are few empirical studies dedicated to study the relation between these specific constructs (academic adaptation and grit). Mendes (2021) found a positive relationship between grit and the perception of learning, academic performance, confidence in completing the course started and the well-being of students. The regression analysis indicated that well-being and the degree of confidence had a significant impact on grit. Furthermore, in Chinese university students, academic adaptability had a negative impact on burnout, that is, the greater adaptability, the lower the level of burnout (Chen et al., 2023). Students with a lower capacity for academic adaptability are more susceptible to psychological stress, academic dropout and less success in their academic journey (Xie et al., 2019; Barroso et al., 2022). Therefore, it is relevant that this variable is studied together with grit, which is a strong predictor of positive academic results (Duckworth et al., 2007; Credé et al., 2017; Allen et al., 2021).

Brazilian university students are known to face a variety of challenges, including academic demands, social pressures and significant life transitions. Consequently, studying constructs such as grit, which helps explain why some individuals are able to reach their full potential (Duckworth et al., 2007); life satisfaction, which reflects how satisfied a person is with their own life (Diener et al., 1997); and academic adaptation, which allows students to adaptively go through the changes imposed on them, can help in understanding student success in academic environments. Studies presented above show that there is a relationship between the variables. This study proposed a mediation model to examine the mediating effect of academic adaptatability in the relationship between grit and life satisfaction following two hypotheses:

*H1*: Grit factors - Consistency of Interests and Perseverance of Effort would directly relate to life satisfaction.

*H2*: Academic Adaptation would serve as a mediator between Grit and Life Satisfaction.

## Methods

### Participants

This study used a non-probabilistic convenience sample with a sample of 413 undergraduated students, 329 (79.70%) were female and 84 (20.30%) male. Ages ranged from 18 to 71 years (*M* = 27.30; SD = 10.20). Of the participants, 90.10% (*n* = 372) were students from private higher education institutions and 9.90% (*n* = 41) from public institutions.

### Instruments

#### Sociodemographic questionnaire

An instrument made up of 11 questions, developed by the authors specifically for this research, with the aim of obtaining data such as gender, age, university and course attended.

#### Grit assessment scale (EAGrIt-LP)

Self-report instrument for assessing grit that was built and validated according to the AERA Standards specifically for the Portuguese language speakers (Noronha and Almeida, 2022). The scale consists of 12 items, distributed into two factors (Consistency of Interests = 6 items and Perseverance of Effort = 6 items): GrIt-LP has a Likert scale response key, ranging from 1 (*Strongly Disagree*) to 5 (*Strongly Agree*). Examples of items are: “I have defined my life goals for the next few years”; “I persevere in my efforts to achieve my goals”; “I try to commit myself to achieving my goals.” In this research sample, the estimated accuracy of the scores was *α* = 0.81 and *ꞷ* = 0.82 for the Consistency of Interests factor, and *α* = 0.84 and *ꞷ* = 0.85 for Perseverance of Effort.

#### Questionnaire on adaptation to higher education (QAES)

This self-report instrument aims to assess the university student’s adaptation process, experiences and academic integration into the higher education institution (Araújo et al., 2014). The instrument was built and validated for the Portuguese language speackers. The QAES consists of 56 items, divided into five factors: Career Planning (6 items), Social Adaptation (12 items), Personal Emotional Adaptation (11 items), Academic Adaptation (8 items) Study Adaptation (12 items) and Institutional Adaptation (7 items). The instrument has a Likert scale response key, ranging from 1 (*Strongly Disagree*) to 5 (*Strongly Agree*). Examples of items are: “Lately at university I’ve felt more irritable than usual”; “Lately I’ve felt disoriented and confused”; “Lately I’ve felt sad or down.” In this study’s sample, the estimated reliability of the scores for the Personal Emotional Adaptation factor was *α* = 0.88 and *α* = 0.89.

#### Life satisfaction scale (SWLS)

A self-report instrument that assesses the cognitive component of subjective well-being - the level of satisfaction of the individuals (Diener et al., 1985). The SWLS has 5 items, which are answered on a seven-point Likert scale, ranging from “strongly disagree” to “strongly agree.” Examples of items are: “My life is close to my ideal,” “My living conditions are excellent”; “I am satisfied with my life.” Zanon et al. (2013) carried out a validation study of the measure for the Brazilian context, gathering evidence of internal structure and realibilty estimates. In this study, the reliability estimates were *α* = 0.83 and *ꞷ* = 0.85.

### Procedures

Initially, the project was submitted to a Research Ethics Committee for appraisal, and was approved under number 5.392.664. Data was collected *online*. The instruments were inserted into the *Google Forms* platform, and the *link to* access the survey was made available on social networks (i.e., *facebook, instagram and whastap*). Participants signed a Free and Informed Consent Form (FICF), which stated the objectives of the research, the instruments used as well as guaranteeing the anonymity and confidentiality of the data. It took an average of 10 min to answer the instruments, in the following order: Sociodemographic and academic characterization questionnaire, EAGrIt-LP, QAES and SWLS.

### Data analysis

Pearson’s correlation analysis was used between the scores on the EAGrIt-LP, QAES and SWLS scales. The values proposed by Cohen (1988) were used to interpret the magnitudes: *r* ≤ 0.29 (weak); 0.30 ≤ *r* ≤ 0.49 (moderate); *r* ≥ 0.50 (strong). We tested a model for investigating the effects of Grit and Academic Adaptation on Satisfaction with Life. Next, the Academic Adaptation as mediating variable between Grit (independent variables) and Satisfaction with Life (dependent variables). The tested models were made through Structural Equational Modeling, with a Maximun Likelihood estimator and bootstrapping of 500 cases. The following fit indices were considered: Root-Mean-Square Error of Approximation (RMSEA; reference value <0.08), Comparative Fit Index (CFI; reference value >0.90) and Tucker-Lewis Index (TLI; reference value >0.90; Hair et al., 2009).

The mediation model was tested following these steps: (a) Grit (independent variable) should had direct effects on life satisfaction (dependent variables); and on academic adaptation (mediating variables); (b) academic adaptation should had direct effects on life satisfaction; (c) the initial effect of Grit on the dependent variable should not be significant or be reduced, since the mediating variables were inserted in the model. We used 95% confidence interval for direct and indirect effects. The analyses were performed using JASP.

## Results

As can be seen in Table 1, the correlation analysis indicated positive associations between the variables, with magnitudes ranging from moderate to high. Consistency of Interests was the factor that showed the greatest association with Academic Adaptation and with Satisfaction with Life.

**Table 1:** Data correlation matrix.

	CI	PE	AA	LS
Consistency of interests (CI)	–			
Perseverance of effort (PE)	0.71	–		
Academic adaptation (AA)	0.40	0.33	–	
Life satisfaction (LS)	0.36	0.33	0.50	–

**Figure 1:**
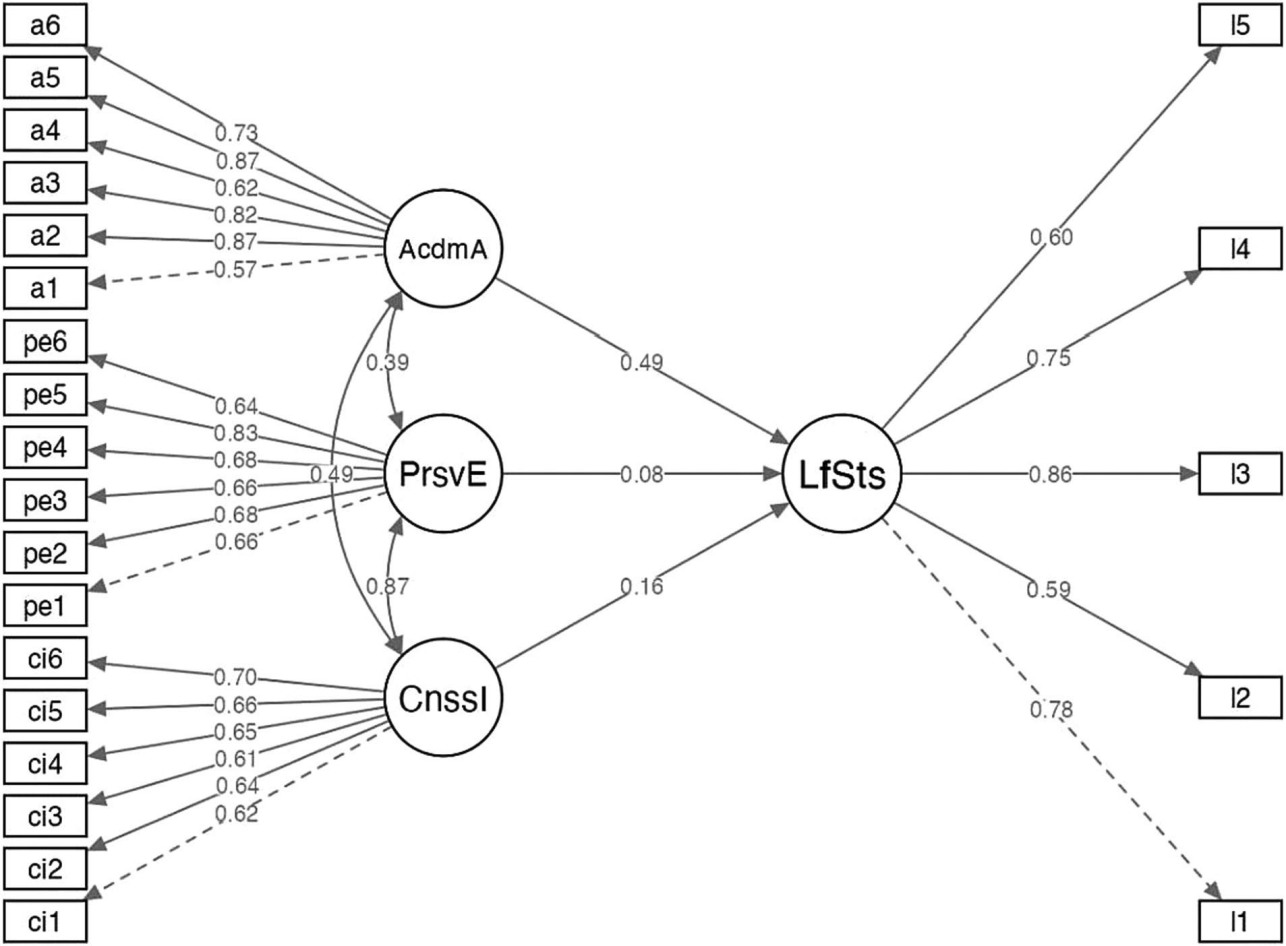
Structure equation modeling of academic adaptation, perseverance of efforts, and consistency of efforts. AcdmA, Academic Adaptation; PrsvE, Perseverance of Efforts; CnssI, Consistency of Interests; LfSts, Life Satisfaction.

To investigate the direct effect of the independent variables on life satisfaction, we performed regression analysis using Structural Equation Modeling. We conducted diagnosis of multicollinearity between the variables. The VIF values obtained ranged from 1.27 to 1.65, indicating that there are no exact or approximately exact linear relations. As shown in Figure 1, Consistency of Interests (B, non-standardized = 0.21; Beta = 0.16) and Perseverance of Efforts (B, non-standardized = 0.11; Beta = 0.08) and Academic Adaptation (B, non-standardized = 0.63; Beta = 0.49) were predictors of Life satisfaction (adjusted *R*^2^ = 0.41), with good model fit indexes (RMSEA = 0.06; CFI = 0.93; TLI = 0.92).

**Figure 2:**
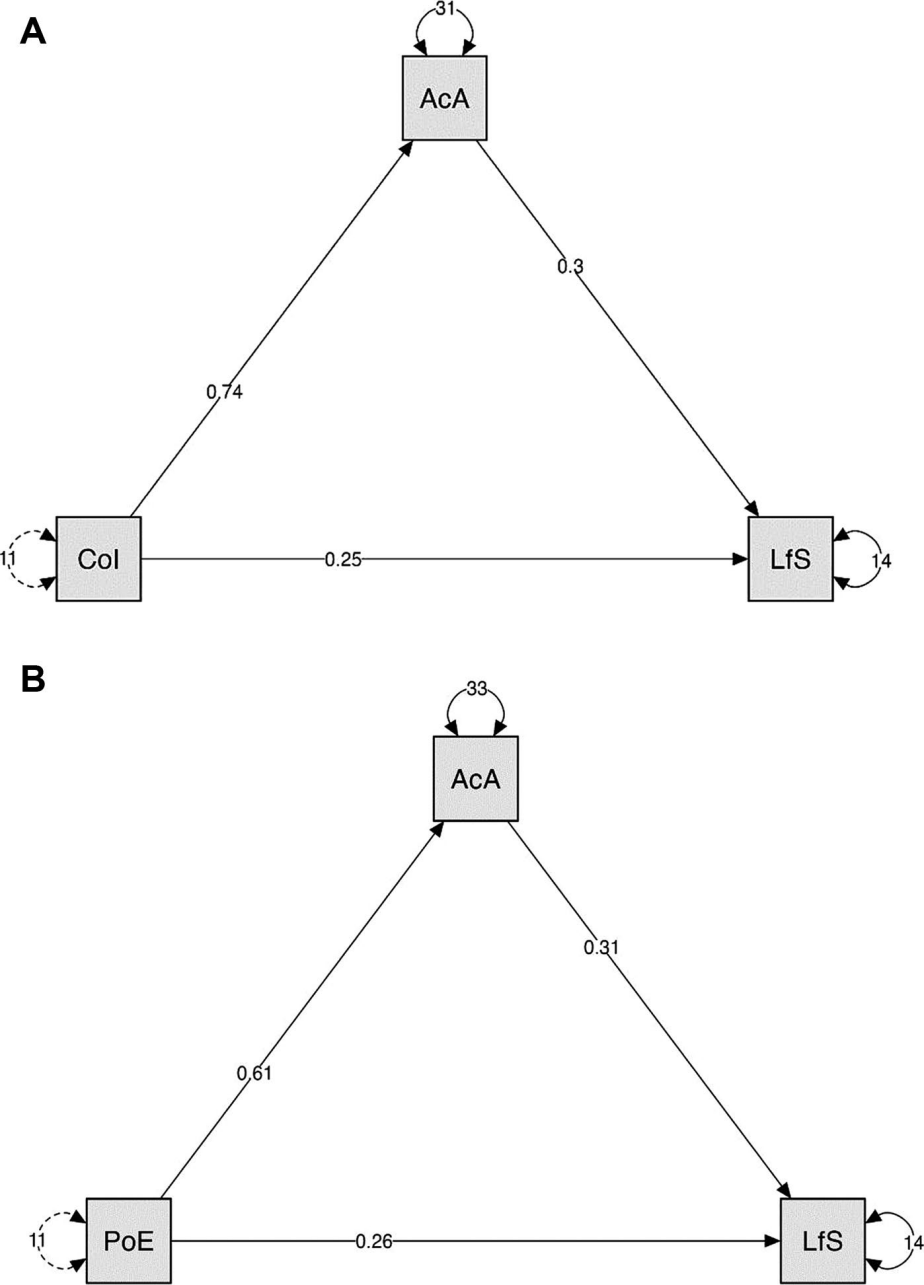
Effect of mediation of academic adaptation on the relationship between grit variables and life satisfaction. AcA, Academic Adaptation; PoE, Perseverance of Efforts; CoI, Consistency of Interests; LfS, Life Satisfaction.

It was possible to observe that academic adaptation mediated the relationship between Consistency of Interests and Satisfaction with Life. The initial direct effect was *b* = 0.47, 95% CI [0.35–0.59], *p* < 0.001 and adjusted *R*^2^ of 0.12. After inserting the mediating variable, the total effect was reduced to *b* = 0.25, 95% CI [0.13–0.37], *p* < 0.001, with an improvement in adjusted *R*^2^ to 0.28. Academic adaptation mediated around 46.80% of the relationship between Consistency of Interests and Satisfaction with Life. Similarly, when Perseverance of Efforts was included as a predictor in the model, the initial effect was *b* = 0.44, 95% CI [0.32–0.56], < 0.001 and adjusted *R*^2^ of 0.11, which was reduced to *b* = 0.26, 95% CI [0.14–0.17], *p* < 0.001 and adjusted *R*^2^ of 0.28. Academic adjustment mediated approximately 40.90% of the relationship between the variables. See Table 2 and Figure 2.

**Table 2:** Standardized effect coefficients of the mediation models investigated.

Model 1		Direct effect without Vmed	Direct effect with Vmed	Indirect effect
Consistency of interests →	Academic adaptation	0.47**	0.25**	0.22**
Academic adaptation →	Life satisfaction			
Consistency of interests →	Life satisfaction			
Model 2				
Perseverance of efforts →	Academic adaptation	0.44**	0.26**	0.19**
Academic adaptation →	Life satisfaction			
Perseverance of efforts →	Life satisfaction			

**p < 0.01.

## Discussion

The present study aimed to analyze the extent to which the relationship between grit and life satisfaction is impacted by academic adaptation. Grit is a trait that informs about the maintenance of interest and perseverance of effort (Duckworth et al., 2007; Duckworth and Quinn, 2009). There is a relationship between grit and life satisfaction that is theoretically justified because people who are committed to their goals tend to be more satisfied (e.g., Khan and Khan, 2017). The question in this study was to assess whether the consistency of interests and perseverance of effort (Noronha and Almeida, 2022) in relation to student satisfaction would be impacted by academic adaptation. The construct is defined as the extent to which the individual is able to develop strategies and deal with stressful situations in order to respond to the challenges imposed by higher education (Almeida et al., 1999).

Adapting to higher education requires a set of provocations portrayed in processes of acquiring academic content inherent to the specifics of the courses chosen, while at the same time requiring the development of skills. Each student brings with them a reality that distinguishes them from their peers, and in addition to academic performance, they are expected to develop marked social skills, adapt to new contexts and rules, incorporate the values of the host institution, and develop new roles that imply autonomy, among others (Tinto, 1993; Soares et al., 2019). It is reiterated in the literature that adaptation to higher education brings benefits to the individual and to society (e. g. Faria and Almeida, 2020; Santos et al., 2022). It is important to mention that, although the academic adaptation instrument currently used generates information on five factors (Career Planning, Social Adaptation, Personal Emotional Adaptation, Study Adaptation, Institutional Adaptation), we only used the one aimed at understanding personal emotional adaptation because it is theoretically justified in relation to the other concepts (Almeida et al., 1999; Almeida, 2007). Findings in the current research corroborate that Grit is directly related to life satisfaction and indirectly related through the partial mediator variable of academic adaptation. The results suggest that the more grit university students have, the more effectively they adjust personally and emotionally to the demands of academic life, which leads to better life satisfaction.

Fernández-Martín et al. (2020) conducted a literature review on grit as a predictor associated with personal, educational and professional outcomes. By analyzing 90 studies, totaling 70,963 participants, the results of the review indicated that people with greater grit have a series of successes in various domains of life, such as better physical and mental health, better interpersonal relationships, better performance at work, better school grades and lower academic dropout. Thus, this series of positive outcomes in a person’s life leads to a state of well-being, which results in better satisfaction with life (Kushlev et al., 2019). Thus, even if stressful events occur, grit, observed in behaviors of passion for interests and perseverance helps the person to achieve their goals, obtaining successful outcomes.

## Conclusions, limitations and future directions

This research shows that, among this sample of Brazilian university students, a large part of the relationship between grit and life satisfaction is explained by academic adaptation. In Brazil, as in other countries where there is evident socio-economic inequality, access to and permanence in higher education includes not only the student’s individual personal and emotional resources, but also financial and social resources. The transition from school to university or entering higher education after years of completing high school, as in the case of older students who are or are not in the job market, demands physical, psychological and financial resources from the student, as well as a commitment to learning academic content and a new set of norms and new models of behavior required by the university (Santos et al., 2022). Faced with this reality, grit and adaptation to the new stage of life are fundamental. Thus, personal and contextual difficulties demand internal mechanisms from the student, such as grit, that through clarity about their life and career goals, persevere in higher education.

The current study examined the mediating mechanism of academic adaptation on grit and life satisfaction. This has two main implications for educators and educational managers. First, the importance of individual characteristics, like Grit, strengthening emotional regulation promoting a growth mindset, and through vicarious experiences. Second, universities, as institutions of human development education, must be committed to transmitting pragmatic content to their students, but they must also be committed to developing soft skills in their students, for the future stage after university and for life (Scheirlinckx et al., 2023). Although the current study contributed to the literature, it still has several limitations to be considered. First, it is important to highlight that the cross-sectional methodological design does not allow to test the causal relationship among variables, requiring that in future studies a longitudinal design be used. Furthermore, it would be interesting to conduct a comparative study according to specific characteristics of the students, such as gender, age, ethnicity, sexual orientation, with the objective of investigating possible differences between the groups.

In particular, concerning future studies, it seems appropriate to test research with an experimental design. An intervention based on the literature could potentially promote higher levels of life satisfaction and adaptation to higher education, both variables investigated in this study, as well as other non-cognitive variables explored by Li et al. (2018), Tang et al. (2019), and Casali et al. (2023). Similarly, analyzing how grit interacts with characteristics inherent to higher education can provide relevant information for the field of knowledge, for managers, and for university students.

## Data availability statement

The raw data supporting the conclusions of this article will be made available by the authors, without undue reservation.

## Ethics statement

The studies involving humans were approved by Universidade São Francisco Ethics Committee. The studies were conducted in accordance with the local legislation and institutional requirements. The participants provided their written informed consent to participate in this study.

## Author contributions

AN: Writing – original draft, Writing – review & editing, Conceptualization, Data curation, Investigation, Methodology, Supervision. JD-V: Writing – original draft, Writing – review & editing, Data curation, Formal analysis, Software. AC: Investigation, Methodology, Project administration, Writing – original draft, Writing – review & editing.
